# Evaluation of detectors for acquisition of pristine depth‐dose curves in pencil beam scanning

**DOI:** 10.1120/jacmp.v16i6.5577

**Published:** 2015-11-08

**Authors:** Christian Bäumer, Benjamin Koska, Jamil Lambert, Beate Timmermann, Thierry Mertens, Patrick Takoukam Talla

**Affiliations:** ^1^ Westdeutsches Protonentherapiezentrum Essen (WPE) GmbH Essen Germany; ^2^ Clinic for Particle Therapy University Hospital Essen, West German Cancer Center (WTZ) Essen Germany; ^3^ IBA Dosimetry Schwarzenbruck Germany

**Keywords:** proton therapy, particle therapy, PBS, pencil beam scanning, integral depth‐dose curves, multilayer ionization chambers

## Abstract

Acquisition of quasi‐monoenergetic ("pristine") depth‐dose curves is an essential task in the frame of commissioning and quality assurance of a proton therapy treatment head. For pencil beam scanning delivery modes this is often accomplished by measuring the integral ionization in a plane perpendicular to the axis of an unscanned beam. We focus on the evaluation of three integral detectors: two of them are plane‐parallel ionization chambers with an effective radius of 4.1 cm and 6.0 cm, respectively, mounted in a scanning water phantom. The third integral detector is a 6.0 cm radius multilayer ionization chamber. The experimental results are compared with the corresponding measurements under broad field conditions, which are performed with a small radius plane‐parallel chamber and a small radius multilayer ionization chamber. We study how a measured depth‐dose curve of a pristine proton field depends on the detection device, by evaluating the shape of the depth‐dose curve, the relative charge collection efficiency, and intercomparing measured ranges. Our results show that increasing the radius of an integral chamber from 4.1 cm to 6.0 cm increases the collection efficiency by 0%–3.5% depending on beam energy and depth. Ranges can be determined by the large electrode multilayer ionization chamber with a typical uncertainty of 0.4 mm on a routine basis. The large electrode multilayer ionization chamber exhibits a small distortion in the Bragg Peak region. This prohibits its use for acquisition of base data, but is tolerable for quality assurance. The good range accuracy and the peak distortion are characteristics of the multilayer ionization chamber design, as shown by the direct comparison with the small electrode counterpart.

PACS number: 87.55.Qr

## I. INTRODUCTION

In particle therapy with pencil beam scanning (PBS), a narrow hadron beam is magnetically steered across the clinical target. PBS is now well established in a number of particle therapy centers, and more centers currently under construction or in planning are being equipped with nozzles which allow PBS beam delivery. In the commissioning phase of a new PBS‐based treatment room, integral depth‐dose curves are obtained for the beam model of the treatment planning system (TPS). The integral depth‐dose curve IDD(z) is the total dose integrated over the whole plane perpendicular to the beam at the depth z.^(1)^ It is measured with a central axis, quasi‐monoenergetic, stationary pencil beam impinging on a large plane‐parallel ionization chamber. These dedicated ionization chambers typically have a radius of several centimeters (i.e., the total dose is measured over a large, but finite, area). The radius of the sensitive volume of the most frequently used plane‐parallel chamber for this purpose, the Bragg peak chamber from PTW (Freiburg, Germany), is 4.1 cm.

It is known that these chambers do not have a 100% geometrical collection efficiency, due to the missing dose contributions beyond the active area of the chamber. This dose ‘halo’ or ‘low‐dose envelope’ is generated by nuclear interactions and single Coulomb interactions of the proton beam in the detection medium (e.g., water) or nozzle^(2)^ leading to a reduced geometrical collection efficiency.^1^, ^3^, ^4^ The contribution from scattering in the medium is most pronounced at intermediate depths of beams with the highest therapeutic energy. It can be mitigated either by correction functions derived from Monte‐Carlo simulations^(2)^ or experimentally. In the current work we pursue the latter approach. In another recent publication, a plane‐parallel chamber segmented into concentric rings has been employed to characterize large‐angle scattering in scanned carbon‐ion therapy.^(5)^ In the work by Lin et al.,^(6)^ which deals with an identical nozzle to that used in this study, the authors present the corresponding lateral dose profiles and show that the ‘halo’ produced in the nozzle is small, albeit not negligible.

In the current study, we investigate the potential benefit of integrating detectors which have a 50% larger radius than the current clinical standard of about 4 cm through measurement of depth‐dose curves. We assess the improvement in the geometrical collection efficiency of a novel large cross section, plane‐parallel chamber which features a 6.0 cm radius active area (brand name ‘Stingray’, IBA Dosimetry, Schwarzenbruck, Germany) over the Bragg peak chamber. The results are compared with the analogous depth‐dose curves acquired under broad field conditions with a 0.5 cm radius plane‐parallel chamber (PPC05, IBA Dosimetry) and corrected for the inverse‐square law. As an alternative to above scanning water phantom measurements, multilayer ionization chambers (MLIC) can speed up data acquisition of depth‐dose curves of clinical particle beams. This has been demonstrated in the past.^7^, ^8^, ^9^, ^10^ Consequently, we evaluate if a large electrode MLIC (brand name ‘Giraffe’, IBA Dosimetry) can replace the combination of an integral chamber and scanning water phantom. For this we compare the depth‐dose curves acquired with a Giraffe to those curves measured with a Stingray. In an analogous manner, depth‐dose curves measured with a Zebra are compared to the corresponding curves measured with a PPC05. The consistency of the range measurements for all measurement techniques is also evaluated.

## II. MATERIALS AND METHODS

### A. Proton therapy system

In the WPE facility, which is based on the Proteus 235 proton therapy system (IBA PT, Louvain‐la‐Neuve, Belgium), protons are accelerated by an isochronous cyclotron up to about 226.7 MeV. In the subsequent energy‐selection system, which consists of a wheel‐mounted wedge, an analyzing magnet system, and slits, the energy can be degraded down to 100 MeV. The beam transport system guides the beam to one of the four treatment rooms.

The measurements were performed using the PBS dedicated nozzle in gantry room 4. The PBS dedicated nozzle^(11)^ comprises of monitor chambers at the entrance and close to the exit, a pair of quadrupole magnets, and a pair of dipole magnets which steers the pencil beam to the desired lateral position. In order to minimize beam broadening, the nozzle is evacuated, except for the vented monitor chambers. The spot sizes, characterized by the full width at half maximum at the isocenter plane, range from 6.6 mm (226 MeV) to 13.4 mm (100 MeV).

The treatment control system allows three modes of beam delivery:

Clinical mode: Treatment is controlled by the oncology information system (OIS) including record‐and‐verify. Electronic communication adheres to the DICOM standard.
2. Standalone mode: This mode is used without the OIS. Fields are defined by a spot list according to a proprietary format. Beam steering and interlock systems are the same as in clinical mode.
3. Continuous Beam Service mode: beam line magnets are tuned by irradiating spots of the desired energy in standalone mode as a preparatory step. Then the user switches to service mode. Here the beam stops are retracted manually to direct a continuous proton beam into the nozzle. Note that in the IBA PBS beam delivery system, the dipole magnets in the nozzle adjust the lateral beam position (see Fig. 1 in Lin et al.^(6)^). This positional correction is switched off in the service mode operation described here. As a result, the beam is not exactly aligned to isocenter.

**Figure 1:**
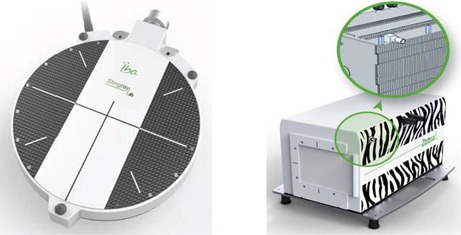
Photo of the Stingray chamber (left), and photo of the Zebra detector (right) and sketch of the inner structure.

### B. Detectors

#### B.1 Stingray chamber

A photo of the detector, which was available as a prototype at the time of testing, is shown in Fig. 1. The electrode radius is 6.0 cm with an electrode spacing of 1.0 mm. The entrance window is fabricated from a carbon fiber composite material. According to the vendor it has a water‐equivalent thickness (WET) for proton beams of 4.2 mm. The Stingray chamber is mounted in the scanning water phantom Blue Phantom^2^ (IBA Dosimetry). For the alignment of the chamber in the phantom there are three alignment tips on the outer rim of the chamber holder. In the alignment procedure of a vertical setup, the positions of the tips are adjusted until all three are just touching the water surface, using two screws in the integrated mechanical holder and one screw on the arm of the Blue Phantom^2^. The sensitive volume of the chamber is then parallel to the water surface at a known water‐equivalent depth z. In our relative dosimetry setup, which is applied also for the Bragg peak chamber, an ‘integrating plane’ of the nozzle monitor chambers IC2/3^(11)^ provides the reference signal. The integrating plane senses ionization for all possible proton trajectories up to the field size limit of 40 cm×30 cm. In the OmniPro‐Accept software (version 7.2 and 7.4b, respectively, IBA Dosimetry), depth‐dose curves are constructed as a ratio of ionization current of the Stingray chamber and reference ionization current.

#### B.2 Bragg peak chamber model 34070

The electrode radius is 4.08 cm with an electrode spacing of 2 mm. The entrance window is 3.5 mm of PMMA, which corresponds to a WET for proton beams of 4.0 mm according to the vendor. The chamber is mounted in a custom‐designed plastic holder in the Blue Phantom^2^, which has plastic screws on the side and at the bottom for fixation and alignment.

#### B.3 Zebra

The Zebra device (IBA Dosimetry) is a commercial MLIC designed to measure depth‐dose curves under broad field conditions.^(10)^ It consists of a stack of 180 independent air‐vented, plane‐parallel ionization chambers fabricated with printed circuit board (PCB) technologies (see Fig. 1). The outer graphite layers of each PCB plate form the circular electrodes which have a radius of 1.25 cm. The air gap between two plates is approximately 1 mm. The average mass density of the PCB plates is close to $2\;g/{\text{cm}}^{3}$ in order to achieve a water‐equivalent device. The material composition of the Zebra/Giraffe is described in Materials & Methods section D below. The effective points of measurements of the 180 chambers constitute a depth axis (z) ranging from about 2 mm to 330 mm (i.e., WET/channel is 1.8 mm–1.9 mm). The ionization charge of each individual chamber is read out by a channel of the electrometer, which is based on Tera06 application‐specific integrated circuit technology.^(12)^ This electronic architecture motivated the term ‘channel’ for a bin on the depth axis as, for example, is plotted in the raw data plot of the acquisition software OmniPro Incline (IBA Dosimetry). The outer dimensions are: 43.9 cm (L)×19.5 cm (H)×17.5 cm (W).. At the start of the operation (e.g., at begin of a shift) of any MLIC device a uniformity calibration is performed. This means that the relative dose reading of each channel is corrected in order to match the corresponding dose reading of a reference measurement in water. For use with proton beams, the smooth proximal section of the Bragg peak is used for calibration. Consequently the highest beam energy available should always be used. In our case 226.7 MeV maximum beam energy allows calibration up to depths of about 29 cm. The WET of a channel, and thus the MLIC depth axis, is established by a linear relation between measured ranges in water (in mm) and measured MLIC ranges (channel number). For the Zebra we used the depth axis calibration commissioned in uniform‐scanning mode in another treatment room.

#### B.4 Giraffe

This large electrode MLIC has the same design as the small electrode version (Zebra), except for its electrode radius of 6.0 cm. Its intended use is to measure the longitudinal depth‐dose distribution of a central axis pencil beam. The depth axis calibration of the Giraffe is set up in the course of this work.

#### B.5 PPC05

The PPC05 plane‐parallel ionization chamber is read out using a Dose 1 electrometer (both from IBA Dosimetry). It features a 9.9 mm diameter collecting electrode and an air gap of 0.6 mm. It has a 1 mm thick entrance window made of C‐552 plastic corresponding to a WET for proton beams of 1.6 mm. Depth‐dose curves under broad field conditions are acquired in point‐by‐point measurements, where each data point originates from an individual application of the field under investigation. According to our experience, the reproducibility of a dose measurement in a time interval of an hour is typically 0.5%.

For the sake of completeness it should be noted that a variable water column also enables the accurate measurements of integral depth‐dose curves.^13^, ^14^, ^15^ It is not tested in the frame of the current study.

### C. Irradiation conditions

Generally, the isocenter was located on the water surface or on the first chamber of the Zebra/Giraffe. Measurements involving a scanning water phantom have mainly been carried out at gantry angle 0° (see Materials & Methods section A). In these measurements, the step size varied between 0.5 mm (in the distal falloff region) to 10 mm (in the entrance plateau) and measurement time was 1 s per data point.

**Figure 2:**
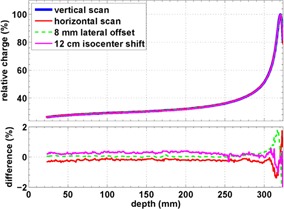
Depth‐dose curves of a 226 MeV pencil beam acquired with the Stingray chamber. A vertical scan along the central axis at gantry angle 0° serves as the reference. Difference of curve shape is computed for the following changes of experimental parameters: scan horizontally, scan exactly on beam axis (which has 8 mm lateral distance from central axis), and isocenter placed at 12 cm depth in water.

The continuous beam service mode has been employed for measurements of integral depth‐dose curves with a scanning water phantom. We checked the possible misalignment with in‐/cross‐line profiles and by placing an EBT3 film (International Specialty Products, Wayne, NJ) on the chamber. A maximum center offset of 8 mm from the surface to the largest depth was measured. For the Stingray chamber, the difference in shape of the depth‐dose curve between irradiation along the nominal axis and irradiation along the actual beam axis was negligible (see Fig. 2). The measured range difference was also negligible, as can also be deduced from geometrical calculations. As a consequence, we acquired the depth‐dose curves on the nominal axis, perpendicular to the water surface.

For measurements with the Giraffe, the beam was delivered at gantry angle 270° in standalone mode.

A $10\;\times 10\;{\text{cm}}^{2}$ quasi‐monoenergetic ("pristine") field is applied in measurements under broad field conditions, as suggested for determination of absorbed dose in the IAEA TRS‐398 protocol.^(16)^ Note that a $10\;\times 10\;{\text{cm}}^{2}$ reference field also matches the optimum irradiation conditions of the Zebra.^(10)^ Fields are delivered in a standalone mode, as described for the Giraffe measurements above. Typically a field application is completed within a few seconds. A lateral spot spacing of 2.5 mm for PPC05 measurements and 1 mm for Zebra measurements produces flat lateral dose profiles.

### D. Calculation of stopping‐power ratio

The Monte‐Carlo software FLUKA^17^, ^18^ version 2011.2b with default settings "HADROTHErapy" has been used to calculate the mass stopping‐power ratios. In our models of Zebra and Giraffe we assume a continuous block of so‐called Zebra‐equivalent material. The inner part of each plate of the Zebra/Giraffe detector is a mixture of Polyimide and Duraver (Isola GmbH, Duren, Germany), where Duraver is a mixture of glass and Polyimide. This structure corresponds to the following stoichiometric composition: O (626), Si (205), B (17), Mg (21), Ca (101), Na (4), Ti (5), H (22), C (961), N (85), Al (61). The specific density is set to 1.054 g/cm^3^ in order to have a WET of about 1.85 mm/channel.

### E. Transforming depth‐dose curves

Divergence of pencil beams of individual spots is the only effect in which source distance is a parameter. As in our PBS delivery system, the divergence is of the order of 3 mrad, the integral depth‐dose curves IDD(z) do not exhibit any dependence on source distance within the clinical positioning range. However, for measurements with a small detector in a 10 cm×10 cm reference field the dose output is subject to a $(z+{\text{ESAD})}^{-2}$ dependence.

The effective source to axis distance (ESAD) is 203 cm in our gantry room 4. We translate depth‐dose curves under broad field conditions M(z) to integral depth‐dose curves with the following equation:

(1) $$IDD(z)=M(z)\frac{{\left(z+ESAD\right)}^{2}}{ESAD^{2}}$$

### F. Comparing depth‐dose curves

The uncertainty of range measurements (distal 80%/90% dose R80/R90) for vertical scans is dominated by the alignment procedure to the water surface. The variability of alignment among physicists gives a precision of ± 0.3 mm. Depending on the length of the measurement, there might be a contribution from water evaporation. In our procedures, we regularly check the surface position of the chamber and correct for changes of water level off‐line after the measurements are performed. We estimate a remaining error of ± 0.2 mm.

There is also a contribution for the positional accuracy of the scanning water phantom. Measurements of the vertical position of the phantom arm in air with a LASER Tracker (model Absolute Tracker 901B, Leica Geosystems AG, Unterentfelden, Switzerland) reveal that the positional error is typically ± 0.1 mm, but can for some depths exceed ± 0.2 mm. Furthermore, the uncertainty in the WET of the chamber entrance wall is about ± 0.2 mm resulting from a typical manufacturing tolerance of the physical thickness of ± 0.1−0.2 mm. So the overall uncertainty is ± 0.5 mm, as shown in Table 1. We refer the reader to Jäkel et al.^(14)^ for further discussions of range uncertainty.

Two depth‐dose curves under comparison are interpolated on a 0.25 mm grid. We decouple the comparison of range from the comparison of curve shape; the depth axis of the second curve is scaled in depth such that the ranges match, thereby eliminating range uncertainties. Depth‐dose curves are scaled in the dose domain to a maximum of 100%. In order to quantify the difference between depth‐dose curves, the dose difference is computed from smallest common depth bin to R80. For assessment of the relative collection efficiency, the ratio of a depth‐dose curve acquired with the two integral chambers of different radii is calculated. This quantity needs normalization, because the effect of the dose halo from the nozzle, which leads to a reduced collection efficiency at water surface, cannot be determined through measurement of depth‐dose curves. Thus, we combined the collection efficiencies at depth z=0 ^(6)^ with the results of the current work.

**Table 1:** Uncertainties of measured ranges

*Uncertainty Component*	*Value (Maximum)*
alignment to water surface	± 0.3 mm
residual error of water evaporation	± 0.2 mm
scanning water phantom positional accuracy	± 0.3 mm
chamber entrance wall WET	± 0.2 mm
total	± 0.5 mm

## III. RESULTS

### A. Stingray chamber in a scanning water phantom

We firstly verified that a vertical scan with the Stingray chamber gives the same shape of the depth‐dose curve as a horizontal scan. For a 226.7 MeV beam (see Fig. 2) and for a 140 MeV beam, the dose difference is less than 1% for depths less than the proximal 80% of the Bragg peak. Secondly, we verified that depth‐dose curves are robust against shifts of the isocenter position in the phantom as expected for a beam line with small divergence (see Materials & Methods section E). Thus, a shift in depth of the isocenter position in the phantom should be negligible. In one of the test runs, the isocenter was placed at 12 cm depth. When compared to the default setting dose, differences are less than about 1%, which are located in the Bragg peak region (see Fig. 2).

**Figure 3:**
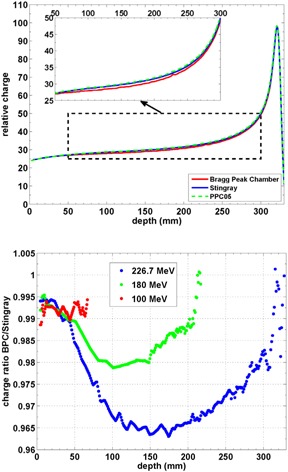
Longitudinal depth‐dose distribution IDD(z) (top) of a 226.7 MeV beam measured with a Bragg peak chamber, Stingray, and PPC05. Ratio (bottom) between the charge measured by a Bragg peak chamber and a Stingray chamber. The comparison has been performed for proton beam energies of 100 MeV, 180 MeV, and 226.7 MeV.

Figure 3 shows an intercomparison of the depth‐dose curves acquired with the Bragg peak chamber, Stingray, and PPC05. The depth‐dose curve for the PPC05 chamber was measured under broad field conditions and has been transformed according to Eq. (1). We show the maximum beam energy of 226.7 MeV as an example, because hadronic interactions have the biggest impact on the shape of the dose distribution. The depth‐dose curve of the Stingray chamber is higher than the corresponding curve of the Bragg peak chamber, but is slightly below the curve of the PPC05 (see Fig. 3 top). The largest deviations between the curves are located in the transition zone between entrance plateau and proximal rise of the Bragg peak.

Figure 3 (bottom) displays the ratio between depth‐dose curves acquired with the Bragg peak chamber and Stingray chamber. The individual depth‐dose curves have been normalized at the water surface to 2.0% (1.2%), 1.3% (0.8%), and 1.3% (0.8%) for 100, 180, and 225 MeV, respectively, for the Bragg peak chamber (Stingray).^(6)^ (L. Lin, personal communication, October 24, 2014.) For 180 MeV, the Bragg peak chamber has a reduced collection efficiency compared to the Stingray chamber of about 2.0% in the worst case, which is at intermediate depth. At the highest energy, the maximum deviation between the ionization chambers is 3.5% (at a depth of about 170 mm). Note that the reproducibility of the relative ionization charge is better than 0.1%, as has been assessed in repeated measurements with the Bragg peak chamber on several days.

### B. Giraffe detector

For uniformity calibration of the Giraffe detector, the plateau region of a longitudinal depth‐dose curve in water is taken (see Materials & Methods section B.). Figure 3 (top) shows that large gradients occur only beyond depths of about 29 cm. Therefore the shallow part of the Bragg peak up to a depth of 29 cm can be used for the uniformity calibration. For the current work, we used the Stingray chamber as a reference for the Giraffe uniformity calibration.

**Figure 4:**
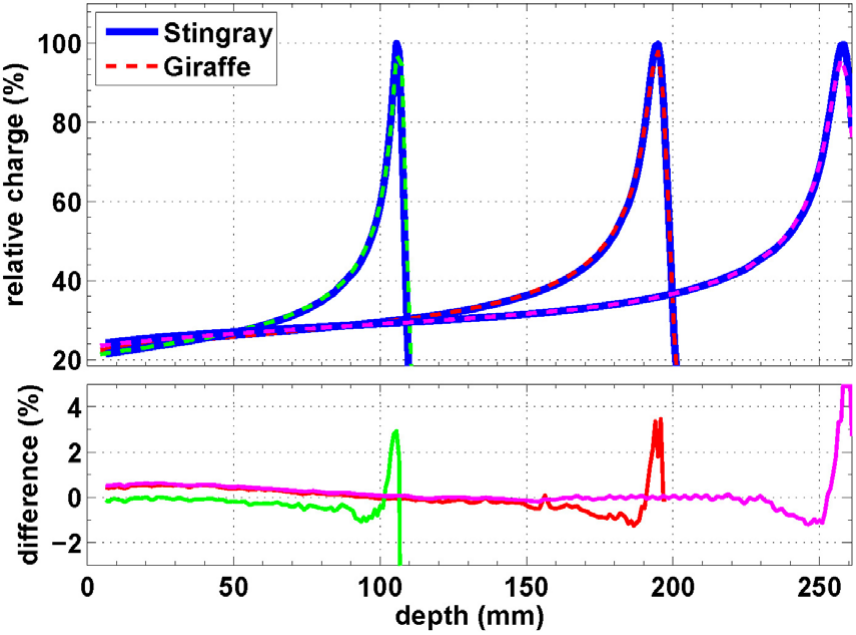
Depth‐dose curves of 120 MeV, 170 MeV, and 200 MeV central‐axis pencil beams; Giraffe measurements vs. Stingray chamber scan in water.

The depth‐dose curve acquired with the Giraffe matches the corresponding water curve, as demonstrated in Fig. 4 for three exemplary beams. We realized that the top of the Bragg peak of a Giraffe acquisition is a bit flatter than in a water scan. For visualization we scaled the relative dose with a factor of 0.97 in order to have matching curve shapes for most parts of the curves. This will be further discussed in the Discussion section B.

### C. Zebra detector

**Figure 5:**
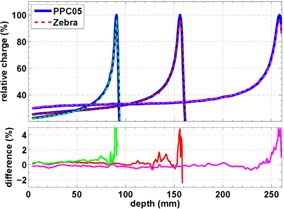
Intercomparison of depth‐dose curves of a 10 cm × 10 cm, 110 MeV/150 MeV/200 MeV field acquired with the Zebra to PPC05 measurement in water.

Figure 5 shows that the shape of a depth‐dose curve of a Zebra acquisition differs slightly from the respective water scan in the same way as for Giraffe. Again, the charge of the Zebra profile has been scaled with a factor of 0.97.

### D. Intercomparison of measured ranges

Table 2 provides an overview of the measured ranges with various methods. We did not apply an identical set of beam energies for all experiments, so the table contains some blanks. There are no measured ranges >29 cm with the MLIC devices as a result of the Zebra/Giraffe calibration procedure. Measurement results in water (columns 2‐4 in Table 2) are from vertical scans.

**Table 2:** Measured ranges (R80)

*Energy (MeV)*	*PPC05 (mm)*	*Stingray (mm)*	*BPC (mm)*	*Giraffe (mm)*	*Zebra (mm)*	*Max. Diff. (mm)*
100.0	78.2	77.8	77.8	77.8	77.6	0.6
110.0	91.8			92.0	91.5	0.5
120.0	107.2	107.4	107.2	107.2	107.2	0.2
130.0	123.1			123.5	123.6	0.5
140.0	140.1	140.2	139.9	140.2	140.3	0.4
150.0	158.2			158.4	158.3	0.2
160.0	177.4		177.3	177.5	177.5	0.2
170.0	197.0	197.2		197.2	197.1	0.2
180.0	217.9	218.3	218.0	218.1	218.0	0.4
190.0	239.2			239.1	239.0	0.2
200.0	261.4	261.1	260.9	261.1	260.8	0.6
210.0	284.5		283.8		283.8	0.7
220.0	308.6					
226.7	325.0	323.9	323.9			

## IV. DISCUSSION

### A. Use of Bragg Peak Chamber and Stingray for commissioning

In the Results section A we assessed the improvement of geometrical collection efficiency if the radius of the integral chamber is increased from 4.1 cm to 6.0 cm. The improvement ranges between 0% and 3.5%, depending on beam energy and depth. The biggest benefit is seen at intermediate depths for all investigated energies. Here the Stingray chamber captures a bigger part of the dose halo which is mainly produced upstream in the detection medium. In the peak region, the collection efficiencies converge and distal data points show a ratio of about 1:1 in Fig. 3 (bottom). This is expected, because off‐axis protons at these depths would exceed the range available for the monoenergetic field under consideration. So a ‘halo’ cannot exist at theses depths. For the remaining multiple Coulomb scattering, it does not matter if the integral ionization chamber has a radius of 4.1 cm or 6.0 cm. This finding corroborates our normalization procedure (Materials & Methods section F) which is based on measurement data from another facility. Data points corresponding to the distal falloff region of the 226 MeV field indicate diverging collection efficiencies. This is regarded as an experimental error, as a ratio is computed in a high‐gradient region.

Suppose that the Stingray chamber is used to generate source data for a beam model of the IBA dedicated nozzle. As indicated in the Materials & Methods section C, these are usually combined with a measurement of absorbed dose in a 10 cm×10 cm field. Figure 3 (top) shows that these measurement conditions are consistent, as the curve shapes of the PPC05 and Stingray deviate only slightly. This holds especially for shallow depths which are suggested for beam calibration of monoenergetic proton fields.^(16)^ Figure 3 (bottom) indicates that measurements miss a part of the contribution from the ‘halo’. Thus, if large field sizes have to be commissioned, the field size for the measurement under broad field conditions in Fig. 3 (bottom) has to be increased (e.g., to 20 cm).

### B. Accuracy of the Zebra/Giraffe depth axis and dose axis

The intercomparison in Table 2 confirms the assumed WET of the entrance windows of the scanned ionization chambers, which are based on vendor information, because there is no systematic range shift between measurements with Bragg peak chamber, Stingray, and PPC05. Furthermore, Table 2 verifies the depth axis of the Zebra, which was established in uniform scanning. The mean deviation to the PPC05 data is less than 0.2%. This justifies the linear relation between channel number and water‐equivalent depth for a Zebra/Giraffe device. Once this depth axis has been cross‐calibrated from water phantom measurements, which has been done on the basis of three independent measurement series in the current work, it does not include the random error for alignment of a single ionization chamber to the water surface and the possible drift due to water evaporation (see Table 1). This eliminates an uncertainty of ± 0.4 mm. Of course, systematic errors of the reference measurement in the water phantom (chamber entrance window thickness and positional error of the depth axis of the water phantom) propagate to the MLIC measurements. The remaining uncertainty is ± 0.4 mm. The observed difference of ranges between Zebra measurements and measurements in a water phantom is in line with Dhanesar et al.^(10)^ That study reports an agreement of range values within −0.1 ±0.4 mm, with a maximum difference of 1.2 mm, for spread‐out Bragg peaks generated by a scattering nozzle.

The normalization of the Bragg peak to 97%, mentioned in the Results section B, might affect the assessment of range. In order to estimate this error, we assume that the top of the Bragg peak is reduced by 3% and that the distal falloff region is undistorted. In this worst case scenario, measured values of R80 would be reduced by 0.15 mm. The Zebra/Giraffe depth axis calibration would consider this shift. So the effect of a possible reduction of the Bragg peak on measured ranges is negligible.

The maximum difference in range for all measurement techniques (last column in Table 2) reflects the uncertainties of the measurement techniques and the reproducibility of the beam line. The latter can be estimated from the monthly constancy checks performed with Zebra over a period of about one year. We observed that the reproducibility of range of the beam delivery system is better than 0.5 mm.

**Figure 6:**
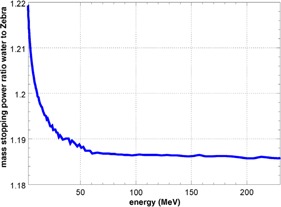
Stopping‐power ratio of water to Zebra/Giraffe‐equivalent material.

The proton stopping power as a function of energy has been extracted from the FLUKA output and compared with the respective values for water. The mass stopping‐power ratio of these materials is shown in Fig. 6. The stopping power changes by about 0.05% in the interval of available beam energies from 100 MeV to 226.7 MeV. Approximating the relation between range and stopping power by a simple inverse equation, we expect a maximum nonlinearity of ranges measured by MLIC detectors of ± 0.06 mm. This corroborates the linear relationship between MLIC channel number and depth in water from a theoretical point of view.

A possible reason for the flattening of the top of the Bragg peak (see Figs. 4 and 5) could be a low‐pass filtering of profiles due to the 1.85 mm sampling steps of the Zebra devices. However, the convolution of the depth‐dose curve acquired in water with a 1.85 mm wide boxcar filter does not yield the observed reduction of Bragg peak height. A possible contribution to the peak reduction is the larger stopping power of water compared to the Zebra/Giraffe for low energies. The rise of the mass stopping‐power ratio with decreasing energy below 50 MeV (Fig. 6) leads to a sharper Bragg peak in water than in Zebra‐equivalent material. Because this effect depends on residual proton range rather than measurement depth, a correction procedure is not straightforward, but is still possible, as described in the work by Sanchez‐Parcerisa et al.^(19)^

### C. Application and limitations of the investigated detectors

We stress that in our center integral plane‐parallel chambers are only used for acquisition of relative depth‐dose curves. As shown in the study by Gillin et al.,^(20)^ they could also be used to measure absorbed dose. Regarding the measurement of relative dose distributions, we identified the following applications for the detectors under investigation:

Bragg peak chamber: is the current clinical standard for acquisition of pristine depth dose curves (e.g., TPS commissioning) or as reference depth‐dose curve for Giraffe (with a maximum energy beam). Due to a limited electrode radius of 4.1 cm, depth‐dose curves can lead to a wrong dose prediction of the TPS^(1)^ if they are not corrected.^(3)^ The dip in efficiency of an integral acquisition in water for a 226.7 MeV beam (see Fig. 3 bottom) translates to a distortion of the measured depth‐dose curve for any measurement with the Giraffe (see Discussion section B).
2. Stingray chamber: has the same application range as the Bragg peak chamber. When used as input for the TPS beam model, there is less need for a correction of the shape of the longitudinal depth distribution. If the reference field for a Giraffe is measured with a Stingray, Giraffe measurements are generally less distorted than a Bragg peak chamber‐based uniformity calibration.
3. Giraffe: is used mainly for quality assurance (QA) of pristine central‐axis pencil beams. For application in QA, it has the advantage over both a Stingray and a Bragg Peak Chamber in that it is fast to set up and does not need a reference signal. Irradiation is possible in clinical mode or standalone mode, in contrast to the scanned chambers which need a service mode in some facilities. In some PBS facilities (e.g., in IBA Proteus centers), this comes with the advantage of a centered beam. Furthermore, for quality assurance application we prefer the clinical or standalone mode, because neither of these requires either special staff training or supplementary experimental checks. The Giraffe detector can, for example, be used to check constancy of range in monthly QA or annual QA. Furthermore, it is ideally suited to measure the water‐equivalent thickness of materials such as immobilization devices, samples of implants, or tables within a several millimeter radius cross section. As an outlook one may note that the Giraffe can be used as a range probe, as described in the study by Mumot et al.^(21)^ In this application, the range of a single spot through beam is measured in order to verify the proton stopping in the patient as calculated in the TPS.
4. Zebra: is used to measure pristine fields and spread‐out Bragg peaks in PBS and passive proton delivery modes.^(10)^

## V. CONCLUSIONS

Depth‐dose curves of quasi‐monoenergetic proton beams have been acquired with novel detectors and compared with well‐established methods. By experiment it was shown that the Stingray chamber with 6.0 cm radius, which was available as a prototype at the time of this study, has an increased geometrical efficiency of up to 3.5% for high therapeutic energies and up to 2.0% for intermediate energies, compared to a chamber radius of 4.1 cm. These numbers refer to the scenario where the halo is mainly produced outside of the treatment head.

For the multilayer ionization chambers, a linear function can be used to transform between MLIC channels and depth in water. These calibrations are sufficient for clinical quality assurance. The user of the investigated commercial MLIC devices has to take care of very small distortions in the dose domain. Consequently, the acquired depth‐dose curves should not be used as input data for a treatment planning system. The large electrode MLIC offers the possibility of a fast quality assurance of pristine beams and can be used as a tool to measure water‐equivalent thickness.

## ACKNOWLEDGMENTS

The authors would like to thank Damien Bertrand, Frédéric Dessy, Gilles Mathot, Andrija Matic, Anne‐Katrin Nix, Peter Wimmer from IBA PT and the WPE physics team for their support.
